# Construction of 2022 Qatar World Cup match result prediction model and analysis of performance indicators

**DOI:** 10.3389/fspor.2024.1410632

**Published:** 2024-11-13

**Authors:** Yingzhe Song, Gang Sun, Chao Wu, Bo Pang, Wuqi Zhao, Rui Zhou

**Affiliations:** ^1^ Institute of Artificial Intelligence in Sports, Capital University of Physical Education and Sports, Beijing, China; ^2^ Department of Physical Education, University of International Business and Economics, Beijing, China; ^3^ Institute of Physical Education and Training, Capital University of Physical Education and Sports, Beijing, China; ^4^ Department of Physical Education, Beijing International Studies University, Beijing, China; ^5^ School of Sports Management and Communication, Capital University of Physical Education and Sports, Beijing, China

**Keywords:** World Cup, football, match performance analysis, machine learning, magnitude-based decision

## Abstract

This research investigates the influence of performance metrics on match outcomes and constructs a predictive model using data from the Qatar World Cup. Employing magnitude-based decision and an array of machine learning algorithms, such as Decision Trees, Logistic Regression, Support Vector Machines, AdaBoost, Random Forests, and Artificial Neural Network, we examined data from 59 matches, excluding extra time. Fourteen performance indicators were integrated into the model, with two types of match outcomes—winning and non-winning—serving as the output variables. The ANN model exhibited the highest predictive performance, achieving an accuracy of 75.42%, an AUC of 76.96%, a precision of 72.73%, a recall of 65.31%, a specificity of 77.03%, and an F1 score of 68.82%. SHAP analysis revealed that “On Target”, “Shooting Opportunity”, and “Ball Progressions” were the most influential features. These findings underscore the critical role of shooting accuracy and the creation of scoring opportunities in determining match outcomes. Consequently, this study developed an accurate model for predicting match outcomes and meticulously analyzed the match performance. Coaches should prioritize the sensitive indicators identified in this study during training and structure training sessions accordingly.

## 1 Introduction

Football performance analysis aims to determine the quantitative relationship among various aspects, links, and components of the system as well as their characteristics by using data to reflect the technical, tactical, and other aspects of the game (1). In other words, it is a research method for investigating the game system. With the rapid development of wearable devices and optical tracking technology, performance analysis has transitioned from simple descriptive statistics to in-depth analysis based on electronic information and artificial intelligence. This technological empowerment promotes the application of artificial intelligence in the development of performance analysis. The fusion of multi source heterogeneous data can facilitate the application of performance analysis in quantitatively studying performance dimensions that were previously difficult to quantify (2). In traditional game analysis research, technical and tactical indicators are often separated from factors such as time, location, and opponents, resulting in a lack of validity and reliability in the construction of technical and tactical evaluation systems. To address this deficiency the data collected for technical, tactical, and physical indicators should cover category, effect, time, location, and defensive intensity aspects (3). As a carrier of technical, tactical, and physical performance information, indicators that reflect the intrinsic and extrinsic characteristics and patterns of the game can guide the team's training and competition (4). The correlation between passes, playing formations, and technical-tactical elements is crucial for understanding team performance during competitions. Offensive formations tend to increase possession and passing accuracy, while defensive formations rely on counterattacks and long passes. High-performing teams also demonstrate better balance in player positioning and pressing strategies, contributing to greater control in key areas of the field (5–7).

With the continuous development of computer science and data mining technology, machine learning algorithms based on artificial intelligence have been proven to predict match outcomes and analyze match characteristics. For example, new supervised models, such as artificial neural networks (ANNs), support vector machines (SVMs), and random forests (RFs) have demonstrated excellent predictive performance in different domains. In recent years, machine learning has been utilized to predict the outcome of sports matches, such as K-nearest neighbors (KNN) algorithm, RF, logistic regression (LR), and SVM (8–10). These models incorporated 9 features and 640 data points, with LR achieving the highest prediction accuracy of 63% (11). Another study applied six different machine learning algorithms (naive Bayes, Bayesian networks, logit boost, KNN, RF, and NN) to predict the results of UEFA Champions League matches, with the NN model achieving a prediction accuracy of 68.8% for win, draw, and loss outcomes (12). In recent years, scholars have used the Bayesian model averaging approach to analyze the relative importance of performance-related factors in determining match outcomes in the “Big Five” European football leagues (English Premier League, German Bundesliga, Spanish La Liga, French Ligue 1, and Italian Serie A) from the 2012/2013 to 2014/2015 seasons. The number of saves made by goalkeepers could be an important factor for predicting team performance; however, it had been overlooked in previous research (13). Besides predicting match outcomes, machine learning can analyze the relationship between indicators and prediction outcomes. For instance, Random Forest (RF) or Decision Tree (DT) models calculate indicators importance using Gini index and information gain (14). SHAP (SHapley Additive exPlanations) is also a powerful and unified metric for interpreting machine learning model outputs. It provides a consistent approach to understanding the impact of indicators on model predictions. This method allows for the fair allocation of each indicator's influence on the prediction, taking into account the potential interactions and dependencies between indicators. Additionally, LIME (Local Interpretable Model-agnostic Explanations) achieves indicators importance analysis by fitting a locally interpretable model around a specific data point (15, 16). These methods offer different perspectives and techniques for interpreting indicators importance, widely used in the explainability research of various machine learning models. Currently, machine learning algorithms commonly used in performance analysis in competitions include ANN, LR, decision trees (DT), RF, SVM, and AdaBoost (17–19). Therefore, selecting more scientific statistical models and inference methods to predict the development trends of tactics and physical demands can improve the decision-making abilities of athletes and coaches, the direction and targeting of training, and the application value of match performance analysis.

Considering the aforementioned points, this study focuses on the 64 matches of the 22nd World Cup as its research subject. By integrating statistical methods and algorithms, such as magnitude-based decision and machine learning, this study explores the impact of competition performance on match outcomes and constructs a predictive model. This study aims to build upon the research achievements of previous scholars and provide a theoretical foundation for coaching practices and enhancing players’ match performance by examining the significance of various dimensions of competition performance in influencing match outcomes.

## 2 Materials and methods

### 2.1 Sample

This study involved the analysis of publicly available data obtained from the post-match analysis reports published by the FIFA Training Center (https://www.fifatrainingcentre.com), and the reliability and accuracy of the data sources in the reports have been validated (20, 21). The total includes 94 indicators related to performance in the competition. Considering the significant difference in data between overtime matches and regular time matches, five matches that entered overtime in the knockout stages were excluded, and the remaining 118 sets of data from 59 matches were analyzed and studied. The dependent variable was the match outcome, and the independent variables were in possession, out of possession, and running-related indicators.

### 2.2 Statistical analyses

#### 2.2.1 Data pre-processing

The possession phase, out of possession phase, and running-related indicators were standardized according to the possession rate of both sides in the match. Among them, the data obtained when the team of interest had possession were standardized to the value corresponding to the team's 50% possession rate:

(1) $${\text{V}}_{\mathrm{standardized}}\text{ = }\frac{{\text{V}}_{\mathrm{original}}}{{\text{P}}_{\mathrm{own}}}\times 50\text{\%}$$

Further, the data obtained when the opponent had the possession were standardized to the value when the opponent had a possession rate of 50%:

(2) $${\text{V}}_{\mathrm{standardized}}\text{ = }\frac{{\text{V}}_{\mathrm{original}}}{{\text{P}}_{\mathrm{opponent}}}\times 50\text{\%}$$

Indicators measured in percentages, such as ball possession rate, shooting accuracy rate, and success rate, were not standardized. Subsequently, nonclinical magnitude-based decision was used to statistically infer the standardized and reciprocal indicators under different game outcomes. Differences in means were converted into effect sizes (ES), and the inferred results were presented as ES ± 90% CI. According to the magnitude of the ES, the ES thresholds for small, moderate, large, very large, and extremely large were 0.2, 0.6, 1.2, 2.0, and 4.0, respectively (22). When the 90% CI for the ES value does not include ±0.2, the difference can be considered pronounced.

#### 2.2.2 Machine learning

Building upon previous research, this study selects several commonly used supervised learning algorithm models in team performance analysis, including DT, Logistic Regression (LR), SVM, AdaBoost, RF, and Artificial Neural Network (ANN) to construct predictive models for match outcomes. These models have their own characteristics and advantages, suitable for different types of data and problems. DT, LR and SVM are widely used supervised learning algorithms in scientific research, such as, predicting match outcomes, a team's goal difference, and players’ physical performance (16, 23–25). AdaBoost and RF are both powerful ensemble learning algorithms widely used in competition performance and spatiotemporal player tracking dataset to predict outcomes or in-game status for their robustness and high predictive performance (24, 26). It reduces the risk of overfitting and enhances the model's accuracy and robustness. ANN is a computational deep learning model inspired by the human brain's neural networks. It can learn complex patterns and relationships in data by adjusting the weights of the connections based on the error in predictions (27, 28). The dataset was split into training (*n* = 106) and validation (*n* = 12) sets while utilizing the 10-fold cross-validation to avoid overfitting the training data (29). The commonly used methods for hyperparameter tuning include Bayesian optimization, random search, and grid search. In this study, grid search was chosen for hyperparameter tuning to automatically select the optimal parameter combination and iterate through the process. The model's evaluation involves the calculation of True Positives (TP), True Negatives (TN), False Positives (FP), and False Negatives (FN) to compute the model's Accuracy (Acc), Precision (P), Recall (R), Specificity (S), and F1 score, as shown in the following formulas:

(3) $$\text{Acc = }\frac{\text{TP + TN}}{\text{TP + TN + FP + FN}}$$

(4) $$\text{P = }\frac{\text{TP}}{\text{TP + FP}}$$

(5) $$\text{R = }\frac{\text{TP}}{\text{TP + FN}}$$

(6) $$\text{S = }\frac{\text{TN}}{\text{TN + FP}}$$

(7) $$\text{F1 = }\frac{\text{2PR}}{\text{P + R}}$$

TP: number of samples predicted as true and their actual values were true, FP: number of samples classified as true but their actual values were false, TN: number of samples classified as false but their actual values were true; FN: number of samples classified as false and their actual values were false (12, 30). Besides The area under the receiver operating characteristic curve (AUC) was calculated to assess the predictive performance of the model. Accuracy, AUC, Recall, Specificity and F1 score explain the predictive performance as follows: 0.5 (meaningless), 0.51–0.69 (poor), 0.7–0.79 (fair), 0.8–0.89 (good), 0.9–0.99 (excellent), 1 (perfect) (31, 32). Considering the role of SHAP values in explaining feature importance, this study selects the model with the highest goodness of fit to calculate SHAP values and analyze their importance on match outcomes (33).

Initially, the raw indicators were standardized, and the effect size for magnitude-based decision was calculated for indicator selection using the Microsoft Excel spreadsheet specially designed by Hopkins (34). Machine learning algorithm models were constructed and competition performance features were analyzed using the Scikit-learn library in Python 3.8.

## 3 Results

**Figure 1:**
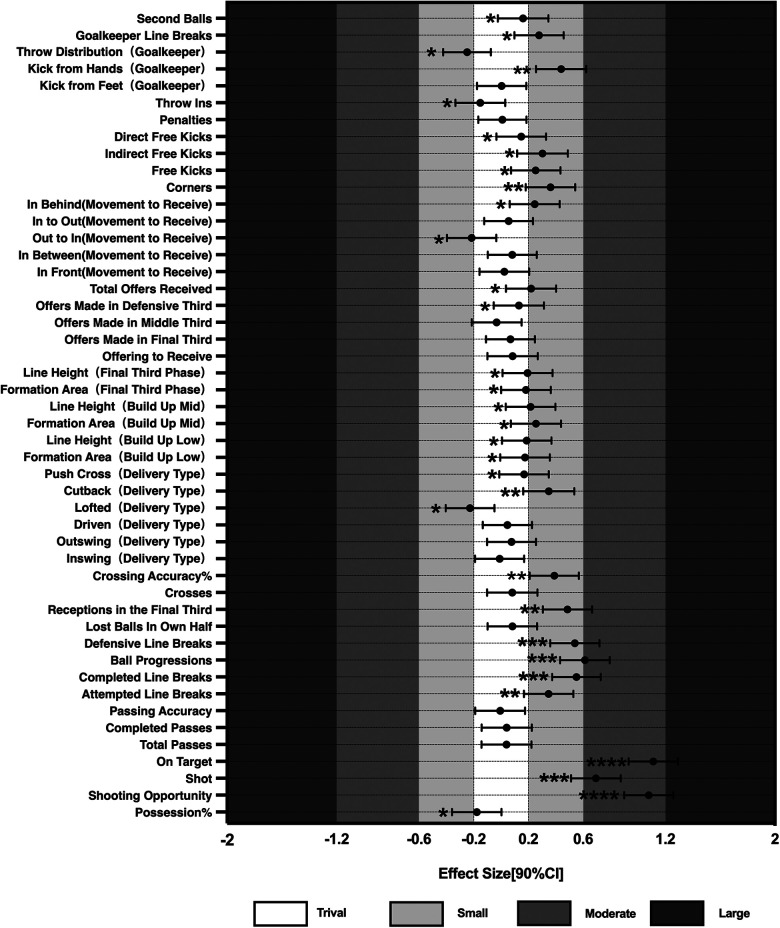
Comparative results of technical and tactical performances in possession phase. When the bars of one variable crossed the negative and positive smallest worthwhile change threshold at the same time, the effect was unclear. Asterisks indicate the likelihood for the magnitude of the true differences between mean as follows: *possible; **likely; ***very likely; ****most likely.

**Figure 2:**
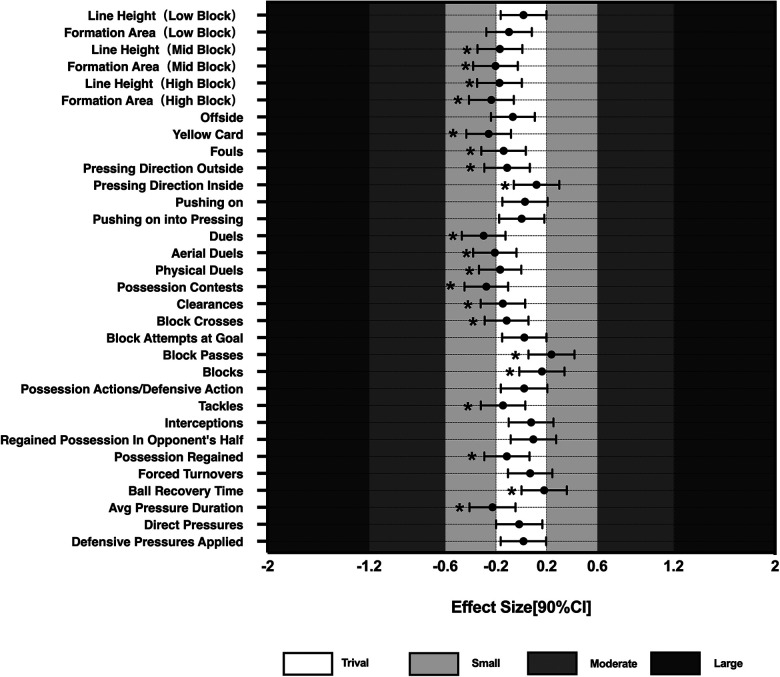
Comparative results of technical and tactical performances in the non-possession phase. When the bars of one variable crossed the negative and positive smallest worthwhile change threshold at the same time, the effect was unclear. Asterisks indicate the likelihood for the magnitude of the true differences between mean as follows: *possible; **likely; ***very likely; ****most likely.

**Figure 3:**
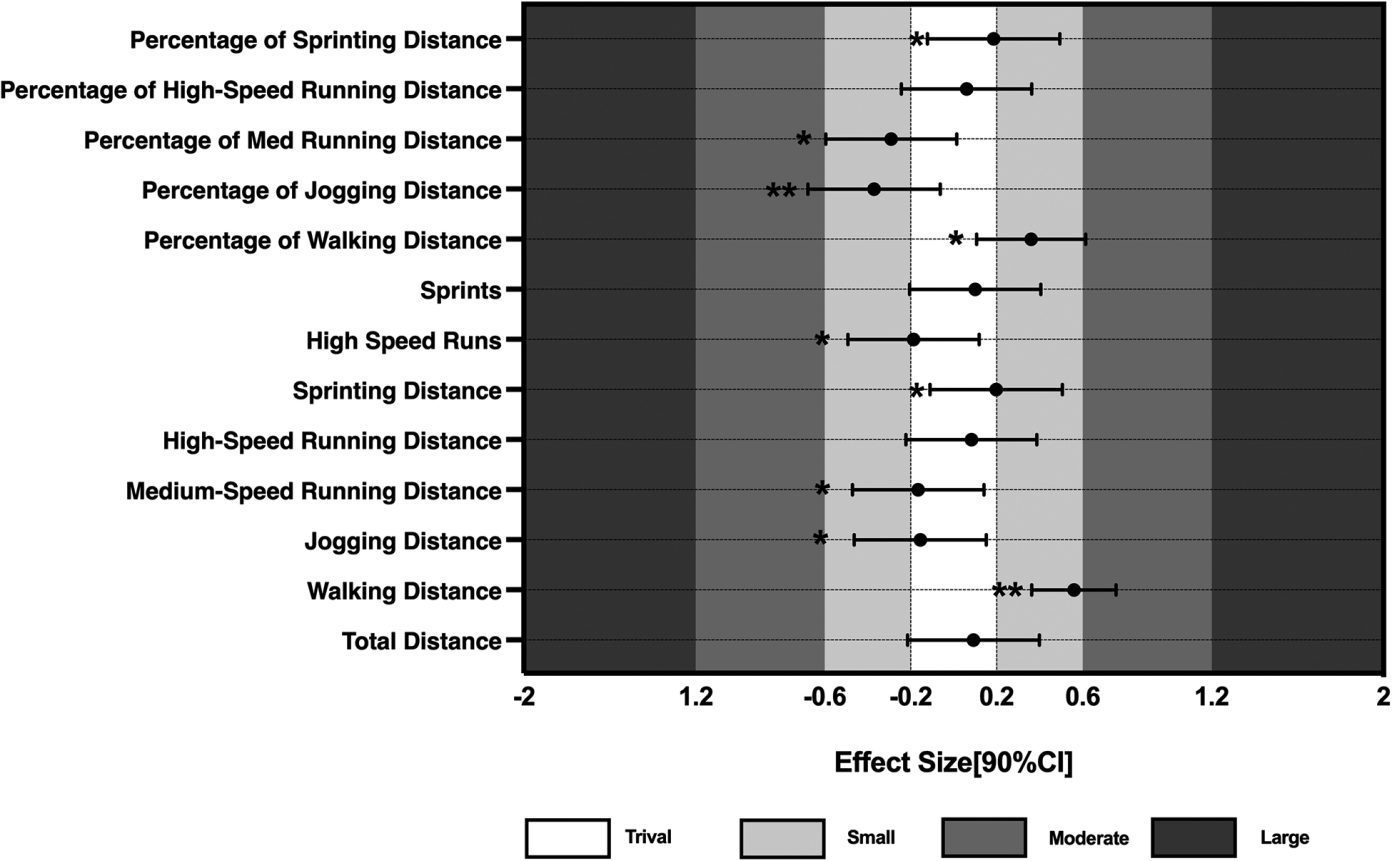
Comparative results of running-related performance. When the bars of one variable crossed the negative and positive smallest worthwhile change threshold at the same time, the effect was unclear. Asterisks indicate the likelihood for the magnitude of the true differences between mean as follows: *possible; **likely; ***very likely; ****most likely.

Magnitude-based decision was utilized to calculate the effect sizes (ES) and confidence intervals of the standardized indicators, concentrating on those metrics that are most likely to impact competition outcomes. The ES values and confidence intervals for the possession phase, non-possession phase, and running-related indicators are presented in Figures 1–3. Fourteen indicators were selected based on the magnitude of their inferred impact on match performance.

**Figure 4:**
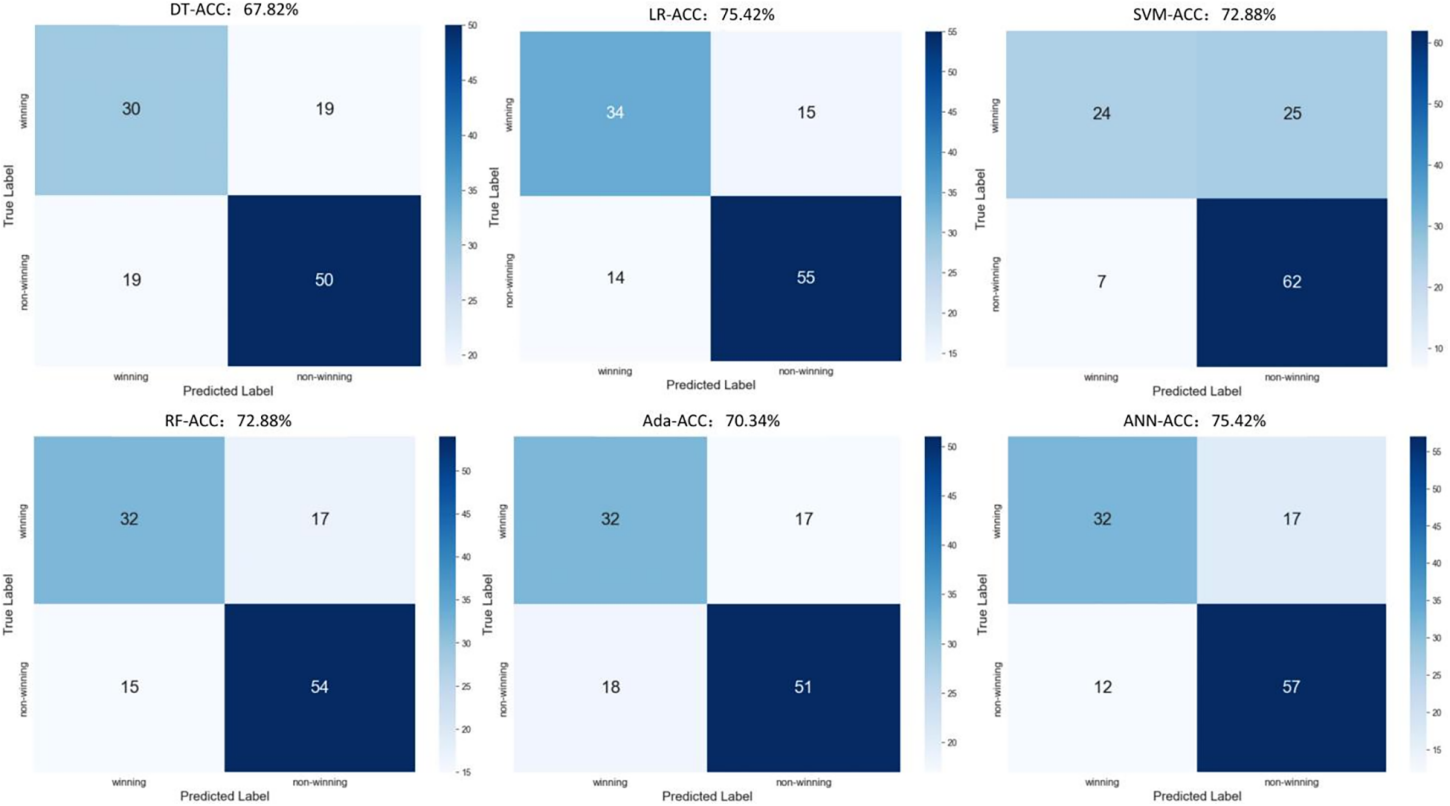
Model confusion matrix.

Table 1 shows the selected input indicators for model construction whereas the outputs were the competition outcomes. The classification of competition outcomes consists of two categories: winning and non-winning, where draws and losses are included in the non-winning category. “Winning” is assigned a value of “0”, and “non-winning” is assigned a value of “1” as the output of the model. Using six different machine learning algorithms to construct a model for predicting competition outcomes, the predictive performances of the different models are shown in Table 2. The confusion matrices of the six models—DT, LR, SVM, RF, AdaBoost, and ANN—are shown separately in Figure 4. By evaluating the accuracy of the predictive models, it is observed that ANN (75.42%) = LR (75.42%) > SVM (72.88%) = RF (72.88%) > AdaBoost (70.34%) > DT (67.82%). However, the AUC value of the ANN model (76.96%) exceeds that of the LR model (74.86%). Overall, the performance of the ANN model is superior in predicting match outcomes.

**Table 1:** Selected indicators.

Categories	Input indicators
In possession	On Target, Shooting Opportunity, Shot, Ball Progressions, Completed Line Breaks, Defensive Line Breaks, Receptions in the Final Third, Kick from Hands (Goalkeeper), Crossing Accuracy%, Cutback (Delivery Type), Corners, Attempted Line Breaks
Running-related	Walking distance, Percentage of Jogging Distance

**Table 2:** Model performance Evaluation.

Model	Accuracy	AUC	Precision	Recall	Specificity	F1 score
DT	67.82%	66.84%	61.22%	61.22%	72.46%	61.22%
LR	75.42%	74.86%	70.83%	69.39%	78.51%	70.10%
SVM	72.88%	78.62%	77.42%	48.98%	71.26%	60.00%
RF	72.88%	74.18%	65.31%	68.09%	76.06%	66.67%
AdaBoost	70.34%	69.65%	64.00%	65.31%	75.00%	64.65%
ANN	75.42%	76.96%	72.73%	65.31%	77.03%	68.82%

**Figure 5:**
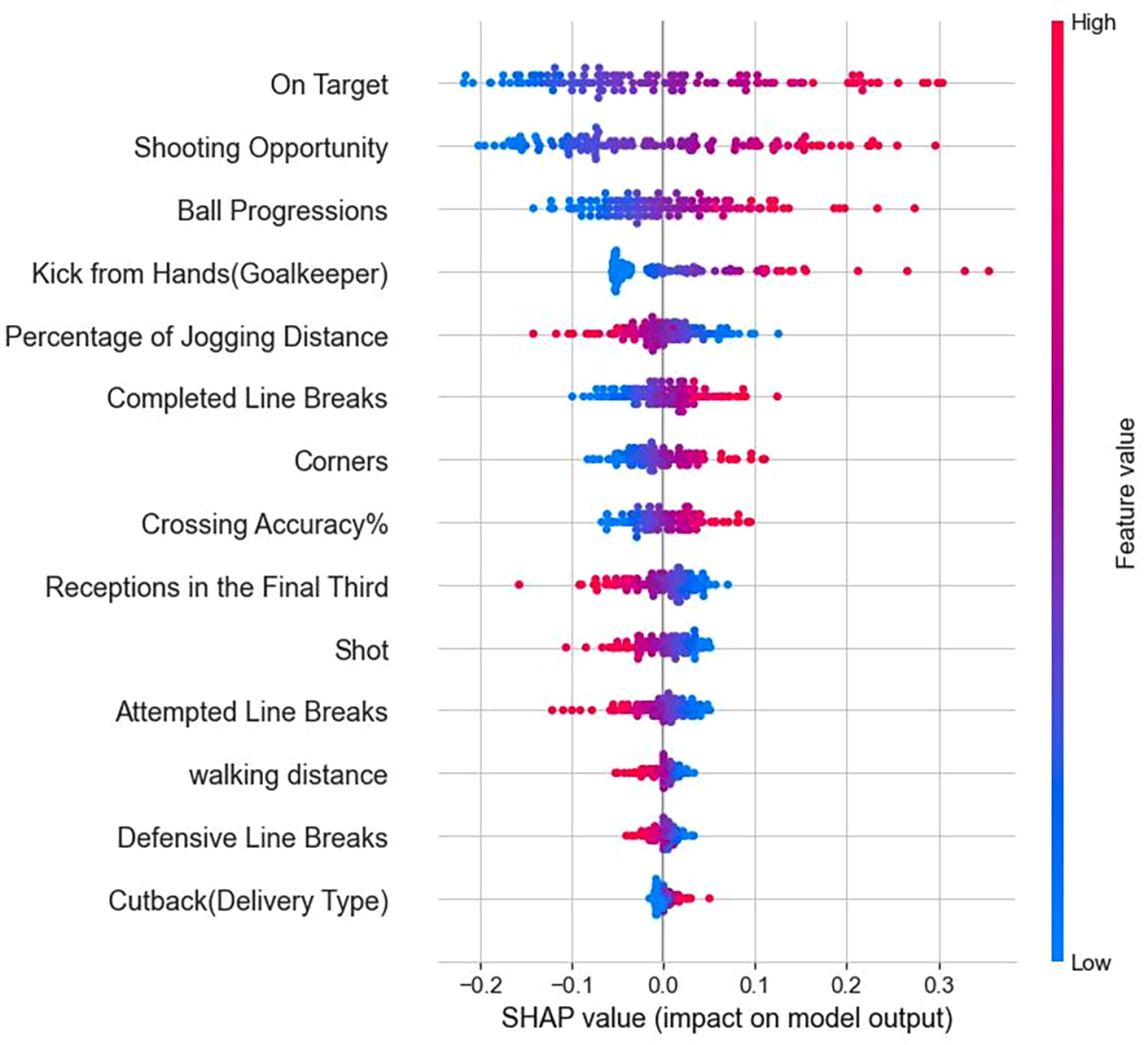
SHAP value ranks.

SHAP values were then utilized to assess the significance of indicators in the ANN model designed for forecasting match outcomes. The importance ranking of the 14 features is shown in Figure 5. SHAP values are on the *x*-axis, indicating the impact of an indicator on the model's output. A positive SHAP value indicates that the feature increases the predicted value, while a negative SHAP value indicates that it decreases the predicted value. The color represents the indicator value; blue dots indicate low significance of the indicators, while pink dots indicate high significance of the indicators.

## 4 Discussion

This study developed a predictive model for match outcomes using performance data from the Qatar World Cup, with the Artificial Neural Network (ANN) model exhibiting the highest predictive performance (Accuracy = 75.42%; AUC = 76.96%; Precision = 72.73%; Recall = 65.31%; Specificity = 77.03%; F1 score = 68.82%). Fourteen indicators were incorporated into the model construction, with their importance ranked as follows: On Target, Shooting Opportunity, Ball Progressions, Kick from Hands (Goalkeeper), Percentage of Jogging Distance, Completed Line Breaks, Corners, Crossing Accuracy%, Receptions in the Final Third, Shot, Attempted Line Breaks, Walking Distance, Defensive Line Breaks, and Cutback (Delivery Type).

Machine learning algorithms have been extensively applied in the realm of team sports. In this study, the ANN model demonstrated superior performance. ANN models are highly effective in capturing non-linear relationships and feature interactions due to their multi-layered architecture. Nonetheless, the Logistic Regression (LR) model achieved a comparable accuracy (75.42%) to that of the ANN model. The linear relationship between competition performance and outcomes may explain why the LR model exhibits strong performance. LR, being a simpler model, is less prone to overfitting compared to ANN, particularly when the dataset is not exceedingly large. Additionally, hyperparameter tuning during model construction can effectively enhance the performance of the LR model. The robustness of the ANN model is reflected in its high precision (72.73%) and specificity (77.03%), indicating its ability to accurately identify non-winning matches. This suggests that the ANN model effectively distinguishes between winning and non-winning conditions, likely due to its ability to process and learn from detailed and varied input indicators (35). In complex scenarios, technical and tactical performance significantly impacts competition outcomes. Therefore, the objective and reliable match outcome prediction provided by the ANN model is more suitable for meeting the analytical needs of match performance than solely relying on expert experience, intuition, or basic statistical data.

From the perspective of predictive performance, our findings exhibit a degree of comparability with previous studies. Some scholars have used the ANN model to predict the outcomes of the 2006 World Cup, achieving an accuracy rate of 76.9%, which is slightly higher than the accuracy rate observed in this study (36). One reason for this difference is that in this study, draws and losses are categorized as non-winning matches, affecting the distribution of the game outcomes dataset. Additionally, the increasing complexity of football matches challenges the predictability of match outcomes (37). When using ANN and LR to build models, the prediction accuracy was 75.04% (38). Using ANN to construct a predictive model for the outcomes of the 2018 World Cup, the model successfully predicted the team's outcomes as either loss or win 72.7% and 83.3% of the time, respectively (39). Furthermore, utilizing MBD for screening performance indicators in competitive settings results in superior predictive performance of alternative algorithmic models compared to previous research (40, 41).

SHAP analysis revealed that the most influential indicators in the ANN model were “On Target”, “Shooting Opportunity”, and “Ball Progressions”. These indicators significantly contributed to the model's predictive accuracy, underscoring their critical role in determining match outcomes. The high SHAP values suggest that frequent occurrences of shots on target and shooting opportunities are strong predictors of match victories. Similarly, effective ball progressions are crucial for creating scoring opportunities, thereby increasing the likelihood of winning. Research has shown that shooting-related indicators, such as the number of shots and shots on target, significantly influence outcomes in various football leagues, including the UEFA Champions League, English Premier League, La Liga, and CSL (42–44). Moreover, these indicators play a crucial role in determining match outcomes under various contexts (45, 46). However, “Shots” exhibit negative SHAP values for higher feature values, indicating a detrimental effect on the model's output. This suggests that winning a match depends more on the quality of shots rather than the quantity (44, 47). In this World Cup, the total number of shots is not the main factor in determining match outcomes; rather, an increase in shots on target improves the probability of winning.

Ball Progressions refer to a player's ability to penetrate the opponent's defensive space through dribbling, thereby disrupting their defensive formation. This concept integrates the player's actions and the defensive strategies employed by the opposing team, thereby granting it spatial attributes for practical implementation. Defenders typically mark their opponents as a defensive method, whereas advancing the ball allows for the penetration of the opponent's defensive territory, creating numerical imbalances and scoring opportunities (48). Completed Line Breaks, Attempted Line Breaks, and Defensive Line Breaks are crucial indicators for predicting match outcomes. Line breaks refer to an attacking player dribbling or passing the ball through the lowest-positioned player in the opponent's defensive line. By counting the number of times the opponent's defensive line is penetrated, the team's attacking style and sequence can be quantified.

In this study, SHAP values revealed that an increase in the number of Receptions in the Final Third decreases the model's predictive accuracy, indicating that winning teams in this tournament were more efficient in their offensive strategies. High values of Corners, Crossing Accuracy%, and Cutback (Delivery Type) enhance the model's predictive accuracy, reflecting that winning teams favor a more direct offensive approach. The importance of corners on match outcomes has been confirmed in major European leagues and in the FIFA Men's and Women's World Cups (49–51). In the 2022 World Cup, 45 goals resulted from crosses, whereas in the 2018 World Cup, only 25 goals were scored from crosses. Dense defense in the middle forces teams to utilize the space on the flanks and create shooting opportunities through crosses. Therefore, winning teams are more efficient in converting crosses into goals than non-winning teams. Research has analyzed the winning factors in the English Premier League, La Liga, and Major League Soccer, finding that crossing is the most crucial passing method in games (12). Teams that are weaker or trailing in score often tend to focus on crossing tactics (52).

SHAP values indicate that Kick from Hands also positively impacts game outcomes. Previous studies emphasized the defensive role of goalkeepers, noting that 21% of their actions focused on controlling space and maintaining possession, while creating scoring opportunities accounted for only about 3% (53). Recent research has found that goalkeepers’ roles in attacking have increased, accounting for more than 75%–80% of their actions, and the quality of their attacks has improved, with a success rate ranging from 88.97% to 91.66% (54). The match philosophies of various countries have unanimously emphasized the importance of transitioning from attack to defense and vice versa. The significance of goalkeepers’ kicks from hands in winning games has confirmed this viewpoint: goalkeepers can be the starting point for transitioning from defense to attack.

This study found no significant differences in high-intensity technical and tactical behaviors between winning and losing teams in the 2022 World Cup. Running indicators, such as high-speed running and sprinting, are no longer effective in predicting match outcomes (55). The percentage of running speeds below 15 km/h and the distance covered at walking were higher in losing teams compared to winning teams. Therefore, it is speculated that the players of the winning team performed better in terms of recovery and the associated lactate clearance after high-intensity exercise (56). Despite the comparable amount of high-intensity activities between winning and losing teams, the potentially slower recovery and running speed of players in the losing teams might predispose them to make more mistakes during the offensive and defensive transition phases. Further studies in this regard to identify the underlying reasons are warranted.

In conclusion, this study developed a predictive model for the outcomes of the Qatar World Cup utilizing the ANN algorithm. It explores the key indicators influencing the outcomes of the Qatar World Cup and summarizes the performance characteristics of both winning and non-winning teams. This provides a theoretical basis for assessing the feasibility of using the ANN algorithm to predict World Cup outcomes.

## 5 Conclusion

The current research findings demonstrate that the ANN model is capable of predicting the outcomes of Qatar World Cup matches with good accuracy. Furthermore, an analysis of the indicators influencing match outcomes was conducted using SHAP values. The most important indicators affecting match outcomes are On Target and Shooting Opportunity, rather than the number of shots. This suggests that in training, more emphasis should be placed on improving the quality of shots and creating shooting space. Ball Progressions and Line Breaks also significantly impact winning matches, and effective attacks should attempt to penetrate the opponent's defense. Crosses and Corners remain crucial offensive tactics for winning teams, and coaches should arrange targeted offensive and defensive training sessions. Winning teams display lower percentages of Jogging Distance and shorter Walking Distances. Additionally, this study found that goalkeepers’ long kicks are a significant method of attack for teams. Therefore, coaches should focus on the sensitive indicators mentioned above during training and arrange sessions accordingly.

## Data Availability

The original contributions presented in the study are included in the article/Supplementary Material, further inquiries can be directed to the corresponding author.

## References

[1] Zhao G Bu Y Zhang L. The progress, problems and tendency of football performance analysis. China Sport Sci Technol. (2014) 50(4):25–32. 10.16470/j.csst.2014.04.009

[2] Zhao G Chen C. Research methods and evaluation index systems of football match performance. China Sport Sci. (2015) 35(4):72–81. 10.16469/j.css.201504009

[3] Yi Q Li YM Zhang MX Cui YX Liu TB Zhang SL Performance analysis: past, present and future. J Shanghai Univ Sport. (2023) 47(2):88–103. 10.16099/j.sus.2022.05.23.0003

[4] Hou HS Zhang L Xia H He F. Discussion and analysis of core winning technical and tactical indicators in football matches analysis on the core indexes of winning technology and tactic of football match. J Beijing Sport Univ. (2013) 36(5):134–9. 10.19582/j.cnki.11-3785/g8.2013.05.026

[5] Michailidis Y Nenos I Metaxas I Mandroukas A Metaxas T. Correlations of passes and playing formations with technical-tactical elements during the 2022 FIFA world cup. J Sports Med Phys Fitness. (2023) 63(12):1309–16. 10.23736/S0022-4707.23.15125-5 37486256

[6] Casal A Maneiro C L R Losada J Iván-Baragaño I. Comparative study of positioning and technical-tactical indicators between teams of different performance levels in the Qatar 2022 FIFA world cup. Kinesiology. (2024) 56(1):101–16. 10.26582/k.56.1.15

[7] Wei X Zhao Y Chen H Krustrup P Randers MB Chen C. Are EFI data valuable? Evidence from the 2022 FIFA world cup group stage. Biol Sport. (2024) 41(1):77–85. 10.5114/biolsport.2024.127382 38188107 PMC10765435

[8] Lock D Nettleton D. Using random forests to estimate win probability before each play of an NFL game. J Quant Anal Sports. (2014) 10(2):197–205. 10.1515/jqas-2013-0100

[9] Perera H Davis J Swartz TB. Assessing the impact of fielding in Twenty20 cricket. J Oper Res Soc. (2018) 69(8):1335–43. 10.1080/01605682.2017.1398204

[10] Baboota R Kaur H. Predictive analysis and modelling football results using machine learning approach for English premier league. Int J Forecast. (2019) 35(2):741–55. 10.1016/j.ijforecast.2018.01.003

[11] Elmiligi H Saad S, IEEE. Predicting the outcome of soccer matches using machine learning and statistical analysis. 2022 Ieee 12th Annual Computing and Communication Workshop and Conference (CCWC) (2022).

[12] Bai L Gedik R Egilmez G. What does it take to win or lose a soccer game? A machine learning approach to understand the impact of game and team statistics. J Oper Res Soc. (2023) 74(7):1690–711. 10.1080/01605682.2022.2110001

[13] Zambom-Ferraresi F Rios V Lera-Lopez F. Determinants of sport performance in European football: what can we learn from the data? Decis Support Syst. (2018) 114:18–28. 10.1016/j.dss.2018.08.006

[14] Iranzad R Liu X. A review of random forest-based feature selection methods for data science education and applications. Int J Data Sci Anal. (2024):1–15. 10.1007/s41060-024-00509-w

[15] Sarmento H Marcelino R Anguera M Campaniço J Matos N Leitão J. Match analysis in football: a systematic review. J Sports Sci. (2014) 32:1831–43. 10.1080/02640414.2014.898852 24787442

[16] Moustakidis S Plakias S Kokkotis C Tsatalas T Tsaopoulos D. Predicting football team performance with explainable AI: leveraging SHAP to identify key team-level performance metrics. Future Internet. (2023) 15(5):174. 10.3390/fi15050174

[17] Tufekci P. Prediction of football match results in turkish super league games. Proceedings of the Second International Afro-European Conference for Industrial Advancement (AECIA 2015) (2016).

[18] Zhang Q Xu HZ Wei L Zhou LQ, ACM. Prediction of football match results based on model fusion. 3RD International Conference on Innovation in Artificial Intelligence (ICIAI 2019) (2019).

[19] Bunker R Susnjak T. The application of machine learning techniques for predicting match results in team sport: a review. Journal of Artificial Intelligence Research. (2022) 73:1285–322. 10.1613/jair.1.13509

[20] Carling C Bloomfield J Nelsen L Reilly T. The role of motion analysis in elite soccer contemporary performance measurement techniques and work rate data. Sports Med. (2008) 38(10):839–62. 10.2165/00007256-200838100-00004 18803436

[21] Castellano J Alvarez-Pastor D Bradley PS. Evaluation of research using computerised tracking systems [amisco (R) and prozone (R)] to analyse physical performance in elite soccer: a systematic review. Sports Med. (2014) 44(5):701–12. 10.1007/s40279-014-0144-3 24510701

[22] Hopkins WG Marshall SW Batterham AM Hanin J. Progressive statistics for studies in sports medicine and exercise science. Med Sci Sports Exerc. (2009) 41(1):3–12. 10.1249/MSS.0b013e31818cb278 19092709

[23] Harrop K Nevill A. Performance indicators that predict success in an English professional league one soccer team. Int J Perform Anal Sport. (2014) 14(3):907–20. 10.1080/24748668.2014.11868767

[24] Dijkhuis TB Kempe M Lemmink KAPM. Early prediction of physical performance in elite soccer matches—a machine learning approach to support substitutions. Entropy. (2021) 23(8):952. 10.3390/e23080952 34441092 PMC8394038

[25] Ivan-Baragaño I Maneiro R Losada JL Arda A. Multivariate analysis of the offensive phase in high-performance women’s soccer: a mixed methods study. Sustainability. (2021) 13(11):6379. 10.3390/su13116379

[26] Lang S Wild R Isenko A Link D. Predicting the in-game status in soccer with machine learning using spatiotemporal player tracking data. Sci Rep. (2022) 12(1):16291. 10.1038/s41598-022-19948-1 36175432 PMC9522646

[27] Ivan-Baragaño I Maneiro R Losada JL Casal CA Arda A. Technical–tactical differences between female and male elite football: a data mining approach through neural network analysis, binary logistic regression, and decision tree techniques. Proc Inst Mech Eng Pt P J Sports Eng Tech. (2024):17543371241254602. 10.1177/17543371241254602

[28] Lee GJ Jung JJ. DNN-based multi-output model for predicting soccer team tactics. PeerJ Computer Science. (2022) 8:e853. 10.7717/peerj-cs.853 35174271 PMC8802790

[29] Hu H Bi X. An empirical analysis of factors influencing the in-game performance of Chinese Olympic champions. J Shanghai Univ Sport. (2023) 47(02):48–59. 10.16099/j.sus.2022.07.05.0009

[30] Delen D Tomak L Topuz K Eryarsoy E. Investigating injury severity risk factors in automobile crashes with predictive analytics and sensitivity analysis methods. J Transp Health. (2017) 4:118–31. 10.1016/j.jth.2017.01.009

[31] Bennett M Bezodis NE Shearer DA Kilduff LP. Predicting performance at the group-phase and knockout-phase of the 2015 rugby world cup. Eur J Sport Sci. (2021) 21(3):312–20. 10.1080/17461391.2020.1743764 32174244

[32] Hopkinson M Nicholson G Weaving D Hendricks S Fitzpatrick A Naylor A Rugby league ball carrier injuries: the relative importance of tackle characteristics during the European super league. Eur J Sport Sci. (2022) 22(2):269–78. 10.1080/17461391.2020.1853817 33210564

[33] Anzer G Bauer P. A goal scoring probability model for shots based on synchronized positional and event data in football (soccer). Front Sports Act Living. (2021) 3:624475. 10.3389/fspor.2021.624475 33889843 PMC8056301

[34] Hopkins WG. Understanding statistics by using spreadsheets to generate and analyze samples. Sport Sci. (2007) 11:23–37.

[35] Şahin M Erol R. Prediction of attendance demand in European football games: comparison of ANFIS, fuzzy logic, and ANN. Comput Intell Neurosci. (2018) 2018:1–14. 10.1155/2018/5714872 PMC6109553 30158960

[36] Huang K-Y Chang W-L. A neural network method for prediction of 2006 world cup football game. The 2010 International Joint Conference on Neural Networks (IJCNN) (2010). p. 1–8. 10.1109/IJCNN.2010.5596458

[37] Rein R Memmert D. Big data and tactical analysis in elite soccer: future challenges and opportunities for sports science. SpringerPlus. (2016) 5(1):1410. 10.1186/s40064-016-3108-2 27610328 PMC4996805

[38] Igiri CP Nwachukwu EO. An improved prediction system for football match result. IOSR J Eng. (2014) 4(12):12–20. 10.9790/3021-04124012020

[39] Hassan A Akl A-R Hassan I Sunderland C. Predicting wins, losses and attributes’. Sensitivities in the soccer world cup 2018 using neural network analysis. Sensors (2020) 20(11), 3213. 10.3390/s20113213 32517063 PMC7309167

[40] Gai Y Volossovitch A Lago C Gómez M-Á. Technical and tactical performance differences according to player’s nationality and playing position in the Chinese football super league. Int J Perform Anal Sport. (2019) 19(4):632–45. 10.1080/24748668.2019.1644804

[41] Yi Q Gómez M-Á Liu H Gao B Wunderlich F Memmert D. Situational and positional effects on the technical variation of players in the UEFA champions league. Front Psychol. (2020) 11:1201. 10.3389/fpsyg.2020.01201 32636779 PMC7318796

[42] Hughes M Franks I. Analysis of passing sequences, shots and goals in soccer. J Sports Sci. (2005) 23(5):509–14. 10.1080/02640410410001716779 16194998

[43] Lago-Penas C Lago-Ballesteros J Rey E. Differences in performance indicators between winning and losing teams in the UEFA champions league. J Hum Kinet. (2011) 27:137–48. 10.2478/v10078-011-0011-3

[44] Liu HY Yi Q Gimenez JV Gomez MA Lago-Penas C. Performance profiles of football teams in the UEFA champions league considering situational efficiency. Int J Perform Anal Sport. (2015) 15(1):371–90. 10.1080/24748668.2015.11868799

[45] Moura FA Martins LEB Cunha SA. Analysis of football game-related statistics using multivariate techniques. J Sports Sci. (2014) 32(20):1881–7. 10.1080/02640414.2013.853130 24742152

[46] Liu HY Gomez MA Lago-Penas C Sampaio J. Match statistics related to winning in the group stage of 2014 Brazil FIFA world cup. J Sports Sci. (2015a) 33(12):1205–13. 10.1080/02640414.2015.1022578 25793661

[47] Lago-Penas C Dellal A. Ball possession strategies in elite soccer according to the evolution of the match-score: the influence of situational variables. J Hum Kinet. (2010) 25(2010):93–100. 10.2478/v10078-010-0036-z

[48] Fernandes T Camerino O Castaner M. T-Pattern detection and analysis of football Players’ tactical and technical defensive behaviour interactions: insights for training and coaching team coordination. Front Psychol. (2021) 12:798201. 10.3389/fpsyg.2021.798201 PMC8685770 34938248

[49] Alves DL Osiecki R Palumbo DP Moiano-Junior JVM Oneda G Cruz R. What variables can differentiate winning and losing teams in the group and final stages of the 2018 FIFA world cup? Int J Perform Anal Sport. (2019) 19(2):248–57. 10.1080/24748668.2019.1593096

[50] Lee J Mills S. Analysis of corner kicks at the FIFA women’s world cup 2019 in relation to match status and team quality. Int J Perform Anal Sport. (2021) 21(5):679–99. 10.1080/24748668.2021.1936408

[51] Prieto-Lage I Bermúdez-Fernández D Paramés-González A Gutiérrez-Santiago A. Analysis of the corner kick in football in the main European leagues during the 2017–2018 season. Int J Perform Anal Sport. (2021) 21(4):611–29. 10.1080/24748668.2021.1932146

[52] Liu H Gómez M-A Gonçalves B Sampaio J. Technical performance and match-to-match variation in elite football teams. J Sports Sci. (2016) 34(6):509–18. 10.1080/02640414.2015.1117121 26613399

[53] Szwarc A Lipinska P Chamera M. The efficiency model of goalkeeper’s actions in soccer. Baltic J Health Phys Act. (2010) 2(2):132–8. 10.2478/v10131-0013-x

[54] Otte F Dittmer T West J. Goalkeeping in modern football: current positional demands and research insights. Int Sport Coach J. (2023) 10(1):112–20. 10.1123/iscj.2022-0012

[55] Konefal M Chmura P Kowalczuk E Figueiredo AJ Sarmento H Rokita A Modeling of relationships between physical and technical activities and match outcome in elite German soccer players. J Sports Med Phys Fitness. (2019) 59(5):752–9. 10.23736/S0022-4707.18.08506-7 29877676

[56] Malone S Mendes B Hughes B Roe M Devenney S Collins K Decrements in neuromuscular performance and increases in creatine kinase impact training outputs in elite soccer players. J Strength Cond Re. (2018) 32(5):1342–51. 10.1519/JSC.0000000000001997 28557851

